# Lung Cancer Screening in Adults: State-of-the-Art and Policy Mapping (2025)

**DOI:** 10.3390/cancers18040596

**Published:** 2026-02-11

**Authors:** Giorgio Firmani, Jacopo Dolcini, Manuela Chiavarini, Pamela Barbadoro

**Affiliations:** 1 Department of Biomedical Sciences and Public Health, Section of Hygiene, Preventive Medicine and Public Health, Polytechnic University of the Marche Region, 60131 Ancona, Italy; 2 Department of Health Sciences, University of Florence, Viale GB Morgagni 48, 50134 Florence, Italy

**Keywords:** lung cancer screening, low-dose computed tomography, early detection, cancer prevention, European Union, non-European Union, public health

## Abstract

**Background/Objectives**: Lung cancer is the leading cause of cancer-related mortality in Europe, and most cases are diagnosed at an advanced stage. Screening with low-dose computed tomography (LDCT) has been shown to reduce lung cancer mortality in high-risk populations, and the 2022 EU Council Recommendation promotes the implementation of structured population-based screening. This systematic review provides a structured, cross-sectional policy mapping of the current landscape of lung cancer screening (LCS) strategies in adults in the 27 EU Member States and 3 non-EU European countries, based on information available up to July 2025. **Methods**: A structured policy mapping was conducted using data collected up to July 2025 from the National Screening Observatory, the WHO, national health authorities, and peer-reviewed literature to assess national LCS policies across 27 EU Member States and 3 non-EU European countries (Norway, Switzerland, and the United Kingdom). **Results**: LDCT-based LCS is implemented in 7 EU countries (25.9%), including one country with an implemented national program (Croatia), one with an authorized national program under rollout (Germany), and five conductive structured pilot programs (Czechia, Poland, Italy, Hungary, and Spain). Among the non-EU countries, 1 of 3 (33.3%), the United Kingdom, has a national program. All initiatives use LDCT as a screening modality, generally with annual intervals. The remaining countries have no organized LCS, although several have announced upcoming pilot studies. **Conclusions**: A progressive expansion of LDCT screening is emerging in Europe; however, significant variability across national policies persists. Harmonized, evidence-based strategies are needed to support broader implementation and ensure equitable access to early detection.

## Simple Summary

Lung cancer is the leading cause of cancer death in Europe, mainly because it is often diagnosed at an advanced stage. Low-dose computed tomography screening (LDCT) can detect lung cancer at an early stage in high-risk individuals, such as current or former smokers, and has been shown to reduce deaths from this disease. In recent years, European institutions have encouraged countries to introduce organized lung cancer screening (LCS) programs, but the level of implementation is different across Europe. This study reviews and compares current LCS policies in European Union countries and other non-European countries. By mapping countries that have introduced screening programs, pilot studies, or no screening at all, this work highlights the gaps and differences in different LCS. The findings can help policymakers, clinicians, and researchers to better understand the current situation and support the development of more consistent lung cancer screening strategies across Europe.

## 1. Introduction

Lung cancer (LC) is the leading cause of cancer mortality in Europe, accounting for over 20% of all cancer deaths annually [1,2,3].

Despite improvements in treatment strategies, overall survival is low because most cases are diagnosed at an advanced stage, when treatment options are limited [4,5], so early diagnosis is a key priority for cancer control strategies across Europe.

Evidence from large-scale randomized trials has shown that low-dose computed tomography (LDCT)-based screening can significantly reduce LC mortality in high-risk individuals. The National Lung Screening Trial (NLST) in the United States demonstrated for the first time a 20% reduction in LC mortality compared with the radiography group [6]. In Europe, the NELSON study confirmed that LDCT is one of the most effective screening methods for reducing LC mortality. Specifically, LDCT enabled a significant reduction in false-positive findings and unnecessary diagnostic procedures without compromising LC detection [7]. Further evidence from European studies, including the Multicentric Italian Lung Detection (MILD) study in Italy, which showed a 39% reduction in LC mortality [8], and the UK Lung cancer Screening (UKLS) study in the United Kingdom (UK), which demonstrated a significant reduction in LC mortality in a random-effects meta-analysis [9]. These studies support the notion that LDCT-based screening can significantly reduce LC mortality when implemented as part of an organized national program. Furthermore, scientific societies such as the European Society of Radiology (ESR) and the European Respiratory Society (ERS) have also approved LDCT-based screening as an effective strategy, highlighting its benefits and harms [5].

In addition to LDCT-based strategies, there is growing interest in molecular and epigenetic biomarkers that can integrate imaging and improve risk stratification for LC screening. Recent systematic reviews and meta-analyses have highlighted the potential role of leukocyte telomere length, DNA methylation signatures, and alterations in mitochondrial DNA copy number as predictors of LC risk [10,11,12].

In response to this evidence, in September 2022, the Council of the European Union updated its cancer screening recommendations to include LC screening for high-risk groups, marking a significant policy shift and encouraging member states to adopt evidence-based approaches [13], in line with other EU initiatives such as Europe’s Beating Cancer Plan and the EU4Health Program [14,15]. Unlike previous EU Council recommendations on cancer screening, which were limited to breast cancer, cervical cancer, and colorectal cancer, the 2022 update is the first policy document at the European level to recognize LCS as a priority to be implemented. However, full-scale, population-based lung cancer screening is not yet widely implemented, even if a growing number of European countries have initiated or are planning pilot programs to assess the feasibility and effectiveness of LDCT-based LC screening.

This study provides a comprehensive review of current LC screening policies and implementation activities across European countries, including both EU Member States and other non-EU European countries.

## 2. Materials and Methods

A structured multi-source search was conducted to collect and synthesize country-level information on lung cancer screening (LCS) policies and implementation strategies based on low-dose computed tomography (LDCT), covering evidence available up to July 2025. The mapping included all 27 European Union (EU) Member States and selected non-EU European countries (United Kingdom, Norway, Switzerland) for which authoritative and sufficiently detailed public documentation could be retrieved. A descriptive analytical approach was used to summarize the current landscape of LCS policies and pilot programs in European countries.

**Data sources and search strategy**. The search was initiated using the National Screening Observatory [16] as a primary entry point and was extended through targeted searches of institutional websites, including the World Health Organization (WHO), national Ministries of Health, national public health agencies, and other official screening or cancer-control program platforms. In parallel, peer-reviewed scientific literature was searched to capture additional contextual information and to cross-check country profiles. The search strategy employed the terms: (Lung Cancer) AND (“screening” OR “early detection”). National screening policies were further investigated through supplementary structured queries via Google and institutional search engines, combining country names with terms such as “lung cancer screening”, “LDCT”, “pilot”, “program”, “recommendation”, and “guideline”.

**Eligibility criteria and operational definitions**. We included documents reporting national or sub-national recommendations, governance decisions, program descriptions, pilots/feasibility initiatives, or official announcements/roadmaps related to LDCT-based LCS. We excluded purely clinical or hospital-level initiatives without a defined screening pathway or public governance endorsement; studies focused exclusively on diagnostic CT outside a screening context; and reports that did not allow for the classification of program status due to insufficient detail.

For each country, LCS status was operationally classified as follows:

- **Implemented (integrated program)**: an organized LDCT screening initiative endorsed by a national/regional health authority and currently delivered as a structured service with a defined eligibility pathway and resourcing (e.g., public funding or formal reimbursement), with population-based or risk-based recruitment/invitation/referral mechanisms and a standardized workflow.
- **Pilot/currently in use (not integrated)**: an organized, protocol-driven initiative actively running in a defined area and/or time period (feasibility/pilot), offering LDCT screening to a defined eligible group, but not yet deployed as an integrated nationwide program.
- **Planned**: an officially announced program or pilot with publicly available documentation (e.g., governmental or institutional statements, roadmaps, or planned start), not yet started or not yet delivering LDCT screening at the time of data cut-off.

**Data extraction and validation**. Data collection was independently performed by two researchers following a standardized protocol. Extracted data were compiled in a structured electronic database (Excel), including current recommendations, program status, target group (age and/or risk factors), test type, screening interval, setting, and funding mechanisms. Each country profile underwent a two-stage validation process: initial double-checking by two independent researchers, followed by final review by a senior researcher.

**Reconciliation of conflicting reports**. When information differed across sources, we applied a pre-specified reconciliation rule prioritizing the most authoritative and most recent documentation. Source hierarchy was: (1) official national/regional health authority documents and dedicated program websites; (2) EU/WHO or other institutional sources; (3) peer-reviewed literature and scientific society statements; and (4) other grey sources. In case of persisting ambiguity, a conservative approach was adopted (e.g., “planned” rather than “pilot”, and “pilot” rather than “implemented”), and “NA” was used when no defensible classification could be assigned. Collected data were subsequently analyzed to compare similarities and differences in LCS availability and recommendations across countries.

## 3. Results

### 3.1. Lung Cancer Screening in EU and Non-EU Countries

The current landscape of lung cancer screening (LCS) programs and policies for adults across European Union (EU) member states and selected non-EU European countries is summarized in Table 1 and Table 2.

**Figure 1:**
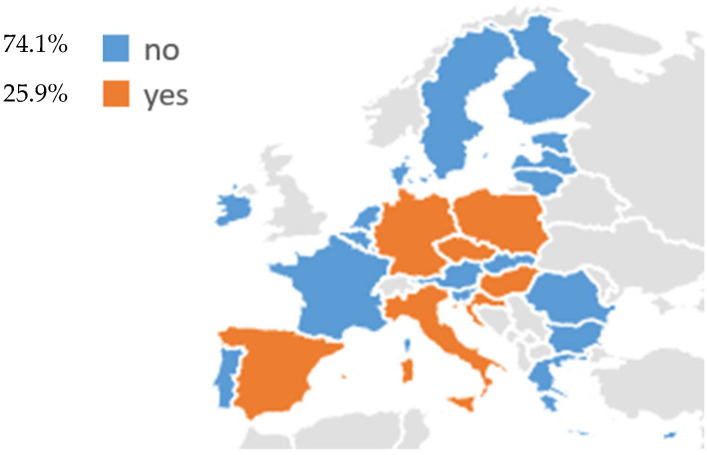
Existing lung cancer screening programs for adults in 27 EU countries.

Regarding EU countries, LCS programs have been identified in 7 EU states (25.9%): Croatia, Germany, Czechia, Poland, Italy, Hungary, and Spain (Figure 1, Table 1). Among these, a fully operational national LCS program is currently implemented in Croatia, while Germany authorized the introduction of a national program in 2024, which is currently being implemented. In addition, the other countries, Czechia, Poland, Italy, Hungary, and Spain, are conducting active national or multi-regional pilot programs. In contrast, most of the EU Member States (20/27, 74.1%), including Austria, Belgium, Bulgaria, Cyprus, Denmark, Estonia, Finland, France, Greece, Ireland, Latvia, Lithuania, Luxembourg, Malta, the Netherlands, Portugal, Romania, Slovenia, Slovakia, and Sweden, have no national LCS program currently in use (Figure 1). Among these, France, Portugal, Slovakia, Sweden, and Greece have announced national pilots, while Ireland has developed research-based or charity-funded initiatives that do not provide an organized LCS system.

**Figure 2:**
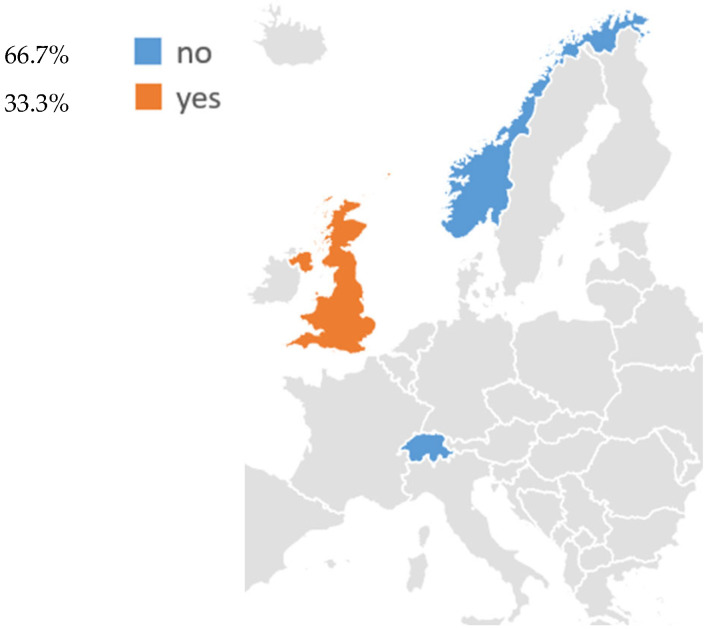
Existing lung cancer screening programs for adults in non-EU European countries.

Regarding non-EU countries, three of these have established or are developing structured LCS activities: the United Kingdom, Norway, and Switzerland (Figure 2, Table 2).

For both screenings organized in EU countries and non-EU countries, LDCT is the only screening modality used in active programs and pilots, with screening intervals generally being annual. Across the 30 countries assessed, explicit eligibility criteria were reported for 15/30 (50.0%). Among these, a lower age threshold was specified in 14/15 (93.3%) and clustered around 50–55 years in 13/14 (92.9%) (50 years in 9/14, 64.3%; 55 years in 4/14, 28.6%), with one broader pilot including ages 18–80 (1/14, 7.1%). An upper age limit was reported in 13/15 (86.7%) and most commonly ranged between 74 and 75 years (10/13, 76.9%). A pack-year threshold was explicitly stated in 9/15 (60.0%), most frequently 20 pack-years (6/9, 66.7%) and less often 30 pack-years (3/9, 33.3%). A former-smoker cessation window was reported in 8/15 (53.3%), most commonly 15 years (6/8, 75.0%), and less often 10 years (2/8, 25.0%). Screening interval was specified in 9/15 (60.0%) and was predominantly annual (8/9, 88.9%), with one targeted program adopting a biennial interval (1/9, 11.1%).

### 3.2. Lung Cancer Screening Recommendations in EU Countries

As above-mentioned, LCS for adults has been identified in seven EU countries, representing 25.9% of member states (Figure 1). These include Croatia, Germany, Czech, Poland, Italy, Hungary, and Spain. Among these, Croatia has fully integrated LCS into its national health system since 2020, while Germany is currently completing the introduction of its national program. In Czech, Poland, Italy, Hungary, and Spain, LDCT-based screening is promoted within publicly funded national or multi-regional pilot programs (Table 1).

In countries where LCS is recommended, authorities recommend LCS for adults aged 50 to 75 with a history of heavy smoking. In particular, national health authorities recommend screening for adults aged 50–75 years in 71.4% of cases (5/7), and 55–74 years in 28.5% (2/7). Eligibility criteria include tobacco exposure ≥20–30 packs/year, which applies to both current smokers and former smokers who quit within the last 10–15 years (Table 1). Notably, France, Portugal, Slovakia, Sweden, and Greece have announced national pilot programs planned for the end of 2025 or 2026; however, these are not yet operational, so they are not considered countries with LCS activated.

Overall, the results indicate that while the LCS rollout in Europe is expanding, it is limited to a minority of EU member states, with differences in the eligible group(s) at risk, funding, and integration within national health systems. Nevertheless, the eligibility criteria and adopted screening comply with the 2022 EU Council Recommendation [13], which presents a new approach to support member states in increasing the uptake of cancer screening, such as for lung cancer.

### 3.3. Lung Cancer Screening Recommendations in Non-EU European Countries

Lung cancer screening initiatives were also identified in three non-EU European countries, such as Norway, Switzerland, and the United Kingdom (Figure 2, Table 2). Among these, the United Kingdom is the only country with an established national program, while Norway and Switzerland are currently conducting pilot research studies.

In the UK, LCS is conducted through the Targeted Lung Health Check program, which started in 2023 and is currently ongoing. This LCS is targeted at high-risk adults aged between 55–74, identified from medical records. This screening is performed every two years, with more frequent follow-ups if lung nodules are detected. The UK represents one of the most advanced LCS with dedicated funding through the National Health Service.

In Norway, there is no population-based screening program; however, several pilot research studies have been conducted. The Norwegian Directorate of Health is evaluating the data collected from the pilot research studies to decide whether LCS should be included in cancer prevention plans.

Finally, in Switzerland, national health authorities have recommended the development of a future pilot program, proposing eligibility criteria that include adults aged ≥50 years with tobacco exposure ≥20 pack-years.

Overall, as in the EU, heterogeneity persists in terms of funding and population eligibility. These findings highlight that LCS is spreading but remains limited to a small number of countries with significant funding for public health

## 4. Discussion

In recent years, the European policy framework for cancer screening has evolved substantially. Building on the Scientific Opinion on cancer screening in the European Union issued by the European Commission Directorate-General for Research and Innovation in 2022 [53], the Council adopted updated recommendations that, for the first time, explicitly include lung cancer among the conditions for which population-based screening should be considered. In parallel, the Joint Action EUCanScreen was launched to support Member States in the sustainable implementation of high-quality organized screening programs for breast, cervical, and colorectal cancers and to investigate the implementation of screening programs for lung, prostate, and gastric cancer [54]. National expert networks, such as the Italian Osservatorio Nazionale Screening, have further disseminated and contextualized these recommendations, highlighting both the opportunities and the organizational challenges linked to the introduction of lung cancer screening in real-world settings [16]. Considering this background, our mapping provides an updated picture of how far European countries have progressed in translating this emerging policy framework into concrete lung cancer screening (LCS) programs and pilots.

In this context, the EU4Health-funded SOLACE project, launched in 2023, aims to support Member States in implementing and optimizing LDCT-based LCS and is expected to develop a European guideline with defined quality standards. The project duration, from 2023 to 2026, suggests that the EU LCS Guideline may become available by the end of the project period [55].

Our results show that only a minority of EU Member States have, to date, implemented some form of organized LCS activity for adults. Seven countries (25.9%), including Croatia, Germany, Czechia, Poland, Italy, Hungary, and Spain, have either fully implemented or are actively piloting LDCT-based screening. Among these, Croatia remains the only country with a fully operational national program already embedded within its national health system, while Germany has authorized a national scheme that is currently being rolled out. The remaining countries are conducting national or multi-regional pilots, often intended to generate real-world evidence on feasibility and implementation, including aspects such as cost-effectiveness, workforce needs, and equity, before moving to full national implementation

In contrast, more than 70% of EU Member States have no LCS program currently in use, despite the common policy direction indicated at the EU level. Within this group, France, Portugal, Slovakia, Sweden, and Greece have announced or planned national pilot programs, and Ireland has developed research-based or charity-funded initiatives, but these do not yet amount to a fully organized LCS system. There are currently no national LCS programs reported in the Baltic countries. However, studies conducted in Lithuania and Estonia have explored strategies for reaching eligible individuals, demonstrating that systematic, population-based approaches can significantly improve screening uptake [56,57]. This pattern illustrates that the pathway from trial evidence to population-based screening is neither automatic nor uniform: it depends on national priorities, health-system capacity, and the perceived balance of benefits, harms, and costs.

A similar, though even more selective, landscape was observed among non-EU European countries. The United Kingdom stands out as one of the frontrunners, having progressed from the Targeted Lung Health Check pilot to a national targeted LCS program funded by the National Health Service. In contrast, Norway and Switzerland are still conducting pilot or research-based initiatives, and their health authorities are using these data to decide whether and how LCS should be integrated into cancer prevention plans [58,59]. Taken together, these examples illustrate a stepwise progression starting from randomized trials to regional or national pilots, and ultimately to fully organized programs that many countries appear to be following, even at different speeds.

Across both EU and non-EU countries where LCS is active or planned, low-dose computed tomography (LDCT) is the only screening modality adopted, with annual or biennial intervals. However, eligibility criteria vary substantially. Most programs target adults aged 50–75 or 55–74 years with a history of heavy smoking, but the precise age ranges and pack-year thresholds (typically ≥20–30 pack-years, with former smokers usually eligible if they quit within the last 10 years) differ between settings. These differences reflect not only diverse interpretations of trial evidence but also local considerations regarding radiology capacity, expected uptake, and acceptable workload for multidisciplinary teams. Such heterogeneity may complicate cross-country comparisons, but it also provides an opportunity to learn from different implementation models, provided that robust monitoring and evaluation frameworks are in place.

An important implication of our findings is that LDCT screening should not be regarded as a narrowly focused intervention for early lung cancer detection only. Evidence from the ITALUNG trial, for example, showed that participation in LDCT screening was associated with decreased cardiovascular mortality, suggesting that the scan can simultaneously capture relevant information on other conditions, such as coronary artery calcification and emphysema, and may provide opportunities to identify comorbid conditions (e.g., coronary artery calcification or emphysema) within a structured pathway; however, the extent to which these translate into broader preventive benefits in routine practice likely depends on program design and requires dedicated evaluation [60]. Moreover, organized LCS provides a unique setting in which to combine structured smoking cessation interventions and other behavioral counseling. In this perspective, LDCT-based programs can integrate secondary prevention of lung cancer and cardiovascular disease with the primary prevention of tobacco-related harm, which may represent additional preventive opportunities; their impact and cost implications should be assessed in dedicated economic evaluations and implementation studies.

Beyond imaging, there is a growing interest in refining LCS eligibility through a more comprehensive characterization of individual risk. A substantial body of evidence indicates that behavioral and environmental factors, such as data-driven dietary patterns and exposure to carcinogens like benzene, can modulate lung cancer risk [61,62]. In parallel, several molecular biomarkers have been proposed as promising tools to capture inter-individual susceptibility, including leukocyte telomere length, DNA methylation signatures, and mitochondrial DNA copy number, all of which have been associated with lung cancer risk in recent systematic reviews and meta-analyses [10,11,12]. Rather than being considered in isolation, these markers are increasingly viewed as complementary to classical risk factors (age, sex, smoking history, comorbidities, and environmental exposures) within multivariable risk-prediction models.

In this evolving framework, integrating behavioral risk factors with molecular biomarkers may provide deeper insights into lung carcinogenesis and, in the longer term, help support LDCT screening through more precise and personalized risk stratification. Such an approach could improve the identification of individuals who are most likely to benefit from screening, potentially optimizing the balance between benefits and harms, with possible implications for cost-effectiveness that should be tested in prospective evaluations. At the same time, the introduction of biomarker-based risk stratification must be carefully evaluated to avoid exacerbating social and geographical inequalities in access to screening and to ensure that any added complexity translates into tangible gains in population health. Nonetheless, at present, evidence remains insufficient for routine population-level implementation, and biomarker-based approaches require prospective validation before being incorporated into organized LCS policies.

Several challenges remain before LCS can be widely and equitably implemented across Europe. Many of the initiatives identified in our mapping are still in the pilot or early implementation phase, and long-term data on adherence, interval cancers, overdiagnosis, stage distribution, and cost-effectiveness in routine practice are limited. The marked heterogeneity of eligibility criteria, organizational models, and funding mechanisms may reflect local needs but also risks, creating a fragmented landscape in which access to screening depends more on country of residence than on objective risk. Ethical and communication issues, such as how to convey the implications of nodule findings, manage incidental findings, and support informed decision-making, require sustained attention, particularly in health systems with constrained diagnostic and treatment capacities.

To translate policy commitments into scalable, high-quality practice, lung cancer screening (LCS) programs increasingly depend on contemporary computational pipelines and trustworthy data infrastructures. AI-based decision-support systems can support LDCT workflows by automating lung and nodule segmentation, prioritizing suspicious scans, and contributing to risk stratification and interval management. Recent lung imaging classification frameworks (e.g., Smart-LungNet) and optimization strategies for deep learning features illustrate relevant technical directions that could interface with screening pipelines, provided that they are externally validated in LDCT screening populations [63,64]. Beyond technical performance, recent evidence syntheses emphasize that real-world deployment requires addressing dataset shift, explainability, workflow integration, and governance/ethical imperatives (e.g., bias, accountability, transparency) [65]. In parallel, secure and interoperable infrastructures, potentially including Internet of Medical Things (IoMT) architectures with blockchain, have enabled audit trails and access control that have been proposed to facilitate privacy-preserving data sharing across screening centers and to support continuous quality assurance and benchmarking [66].

Importantly, these technical tools also offer concrete pathways to mitigate heterogeneity in eligibility criteria and implementation models. AI-assisted risk stratification may help harmonize eligibility definitions by integrating clinical risk factors (e.g., age, smoking history and intensity, comorbidities) with imaging-derived features, supporting more consistent targeting of high-risk individuals across regions and potentially improving the benefit/harm balance. Moreover, multimodal integration of clinical data, LDCT features, and emerging biomarkers may reduce variability in downstream management, allowing for more uniform follow-up strategies across programs, although prospective evaluation and robust governance remain prerequisites for routine adoption [65]. Finally, while deep learning dominates current research, established machine-learning approaches (e.g., gradient boosting and support vector machines) remain useful baselines for transparent benchmarking and external validation when implementing decision support in population-based contexts [67]. Collectively, these considerations suggest that harmonization in LCS is not only a policy challenge, but also a data and infrastructure challenge, for which technical solutions can be aligned with organizational governance and quality assurance.

A exhaustive approach of LC policy screening should also consider several aspects beyond the only cancer screening aim. First of all, contemporary LCS programs must also operationalize the management of incidental findings detected on LDCT (e.g., emphysema, bronchiectasis, interstitial lung abnormalities, coronary artery calcification, mediastinal or thyroid abnormalities), which can provide preventive opportunities but may also increase downstream testing, anxiety, and costs if pathways are not predefined. A European multi-society statement has proposed evidence-based principles for reporting and managing incidental findings in LCS to minimize harm and preserve program cost-effectiveness, supporting structured reporting and clear thresholds for action and referral within screening governance [68]. Second, a detailed economic evaluation should be taken into consideration when applying LCS, since moving from pilots to sustainable population-based LCS requires health-economic evaluation and resource planning (radiology workforce, CT capacity, smoking cessation integration, IT infrastructure, and quality assurance). Cost-effectiveness is sensitive to program design choices, including eligibility criteria, adherence, nodule management protocols, and the handling of incidental findings; therefore, economic modeling should be considered an integral component of “state-of-the-art” implementation rather than an add-on, particularly when scaling to national and cross-national settings. Finally, the eligibility criteria represent a fundamental aspect in the context of a complete and exhaustive scenario for implementing LCS since it can affect both cancer and incidental findings and economic aspects: although most European implementations currently target high-risk individuals primarily defined by smoking exposure, lung cancer in never-smokers and persistent inequalities in access and outcomes raise important implementation questions. Recent analyses caution against routine LDCT screening in never-smokers without robust evidence of net benefit, highlighting overdiagnosis and potential harms and the need for better risk-prediction approaches to identify truly high-risk subgroups. At the same time, targeted “lung health check” models and equity-oriented delivery strategies (e.g., outreach in deprived areas) are increasingly recognized as key enablers to maximize uptake and avoid widening disparities as screening scales up [69,70,71].

This study has some limitations. The information summarized in Table 1 was derived from the published literature, official documents, and grey sources. Despite our systematic approach, some programs or pilots may have changed status or may not yet be publicly documented. Furthermore, the use of grey literature and publicly available reports may have introduced reporting bias, as the quality of documentation can differ between countries. We did not conduct formal health-economic modeling, workforce capacity assessments, or outcome evaluations; therefore, considerations on cost-effectiveness, capacity, and broader preventive opportunities are presented as interpretative implementation issues informed by the literature. The policy environment is highly dynamic: initiatives like EUCanScreen and the 2022 Council Recommendation are likely to accelerate decision-making over the next few years, meaning that the current snapshot will need regular updating. Nonetheless, our findings provide a structured baseline against which future developments can be monitored and highlight the importance of transparent, comparable reporting on LCS implementation across Europe.

## 5. Prospects

Artificial intelligence (AI) is increasingly proposed to enhance multiple steps of LCS, from pre-screening risk stratification to LDCT acquisition, image interpretation, and longitudinal surveillance. In high-volume settings, AI can support reader triage and standardization, potentially reducing false positives and inter-reader variability, and may also enable multimodal integration with clinical variables and emerging biomarkers. Recent works illustrate the breadth of approaches, from deep-learning architectures for automated lung imaging classification (e.g., Smart-LungNet) [63] to optimization strategies (e.g., genetic algorithm-optimized deep learning features) that aim to improve performance while reducing dimensionality and computational burden [64]. A recent systematic review synthesized technical breakthroughs and highlighted key clinical barriers and ethical imperatives for implementation, including explainability, bias mitigation, regulatory compliance, and the need for prospective, externally validated evidence before routine clinical adoption [65].

These innovations are tightly coupled with data infrastructures. Large-scale screening programs require secure and interoperable platforms to store LDCT images, structured reports, and longitudinal outcomes, enabling audit, quality assurance, and potential model updating over time. Emerging proposals combine Internet of Medical Things (IoMT) architectures with blockchain-based mechanisms to strengthen data integrity, access control, and traceability in distributed healthcare environments [64]. While deep learning approaches dominate many recent pipelines, established machine-learning classifiers (e.g., gradient boosting and SVM) remain common and can provide useful baselines for benchmarking and external validation [67].

Finally, alongside imaging-centered innovation, biomarkers remain an active research area with potential to complement LDCT by refining risk stratification and supporting clinical decision-making. Blood-based biomarkers such as plasma microRNA, circulating tumor DNA, and autoantibodies are being investigated to enhance early detection and mitigate false positives, although substantial work remains to validate their clinical utility at the population level and to integrate them into screening pathways.

An additional important element to be considered in the context of future prospects aimed to improve LCS is that, beyond national policy adoption, the success of organized LDCT-based lung cancer screening (LCS) depends on delivery models that support informed participation. Primary care can play a pivotal role in communicating the potential benefits and harms of screening, facilitating shared decision-making, identifying eligible individuals (including those who may not proactively seek screening), and integrating smoking cessation pathways within a “lung health” approach [72]. In this perspective, primary care may also contribute to longitudinal risk assessment and to the timely coordination of diagnostic pathways following abnormal findings, aligning screening implementation with broader developments in lung cancer diagnostic strategies [73]. However, informed participation alone is not sufficient if access remains unequal across communities. Equity-oriented implementation also requires proactive outreach strategies to reach populations and geographic areas with persistently low screening uptake. In this context, radiology-led or multidisciplinary outreach (e.g., coordinated “lung health” initiatives, partnership with primary care and community services, and targeted invitation pathways) can help reduce disparities by focusing on underserved neighborhoods and areas identified through area-level socioeconomic indicators. Although the specific operationalization may vary across health systems, the use of geospatial approaches (e.g., postal-code/area-level targeting) and the explicit integration of social determinants of health into screening interventions have been highlighted as key components to improve participation and equity in cancer screening programs [74]. Finally, when considering the advantages of organized LDCT-based LCS, it is important to frame potential benefits alongside the healthcare workload required for safe implementation at scale. Earlier detection may increase the proportion of patients diagnosed at potentially curable stages and can support more timely, effective management pathways; however, it also generates additional downstream activity (e.g., repeat imaging, diagnostic work-up of indeterminate findings, and follow-up care), with implications for radiology capacity, workforce, and quality assurance. Consequently, the overall value of screening depends not only on clinical effectiveness but also on system readiness and cost-effectiveness under real-world conditions. A relative recent, umbrella review synthesizing evidence from systematic reviews on the cost-effectiveness of lung cancer screening and related strategies highlighted that economic conclusions vary across settings and assumptions (e.g., eligibility criteria, participation/adherence, diagnostic pathways, and unit costs), supporting the need for context-specific planning when scaling national and cross-national programs [75].

## 6. Conclusions

In conclusion, LCS in Europe is expanding but remains restricted to a relatively small number of countries, with substantial heterogeneity in eligibility criteria, funding structures, and degree of integration into national health systems. LDCT has emerged as the standard screening modality. Although some programs may also include cardiovascular risk assessment and smoking cessation, these represent potential additional benefits rather than a single-disease intervention [55]. Looking ahead, the integration of behavioral risk factors and molecular biomarkers into risk prediction models is discussed in the literature as a potential avenue for improving the precision and equity of LCS, but such innovations are not currently reflected in national policies. Overall, our findings underscore the descriptive landscape of LCS implementation and the need for coordinated assessment and policy development across Europe.

## Figures and Tables

**Table 1:** Lung cancer screening recommendations in EU countries.

EU Country	LCS Fully Integrated in NS	Years LCS Fully Integrated in NS	LCS Currently In Use	Eligible Group(s) at Risk	Intervals Test of Screening	Funding	References
Austria	No	-	No (pilot projects planned). Authorities recommended the implementation of LCS	-	-	-	[17]
Belgium	No	-	No (no national program; NELSON ^1^ trial completed)	-	-	-	[18]
Bulgaria	NA	-	-	-	-	-	[19]
Croatia	Yes (national program)	2020–present	Yes (nationwide LCS implemented)	Ages 50–75; heavy smokers (current/former, quit ≤ 15 yrs ago)	Annual	Public (National Health System)	[20]
Cyprus	No	-	Implementation of the LCS program in the near future.	-	-	-	[21]
Czechia	Yes (population pilot program)	2022–present	Yes (5-year national pilot ongoing)	Ages 55–74; ≥20 pack-year smokers (current/former)	Annual	Public (government-funded pilot)	[22,23]
Denmark	No	-	No	-	-	-	[24]
Estonia	No	-	No (LCS has been piloted in one county)	-	-	-	[25]
Finland	No	-	No (ongoing regional pilot study that integrates smoking cessation initiatives with LCS)	-	-	-	[26]
France	No (pilot program planned)	-	IMPULSION ^2^: planned national pilot (start in late 2025)	Ages 50–74; heavy smokers (current/former, ≥20 pack-years)	Annual (planned)	Public (Ministry of Health)	[27]
Germany	Yes (authorized, in rollout)	2024–present	Yes (nationwide program in development)	Ages 50–75; heavy smokers >25 years, quit ≤ 10 yrs ago	Annual	Statutory health insurance (pending G-BA ^3^ approval)	[28]
Greece	No	-	No (no national program; one pilot study completed)	Pilot: smokers 18–80, >20 pack-years, ≥15 pack-years	-	-	[29]
Hungary	Yes (pilot programs)	-	Yes (three pilot programs: HUNCHEST-I ^4a^: 2013–2020; HUNCHEST-II ^4b^: 2019–2022; HUNCHEST-III ^4c^: 2023–ongoing)	High-risk smokers (Age. HUNCHEST-I ^4a^: 50–79; HUNCHEST-II ^4b^: 50–75; HUNCHEST-III ^4c^: 50–75)	Annual (in pilots)	Public (national pilot programs)	[30,31]
Ireland	No	-	Yes (pilot: Lung Health Check, launched ~ 2023)	High-risk participants (current/former smokers, invited via general practitioner)	-	Charity/Research (Irish Cancer Society & EU SOLACE ^5^)	[32]
Italy	Yes (pilot RISP)	-	Yes (pilot RISP ^6^ program in 18 centers)	Age 55–75; heavy smokers ≥ 30 pack-years (current or quit < 10 yrs)	Annual	Public (Ministry of Health pilot)	[33]
Latvia	NA	-	-	-	-	-	[34]
Lithuania	NA	-	-	-	-	-	[35]
Luxembourg	NA	-	-	-	-	-	[36]
Malta	No	-	Implementation of the LCS program in the near future.	-	-	-	[37]
Netherland	No (only one simulated screening based on data from NELSON ^1^ and NLST ^7^ trials)	-	No (no national program; NELSON ^1^ trial in Netherlands/Belgium showed mortality benefit)	Simulated screening: Age 50–74; high-risk smokers (≤30 pack-yrs, quit ≤ 15 yrs)	Annual	-	[38,39]
Poland	Yes (pilot program named WWRP ^8^, which will support the implementation of a national screening program in late 2025)	First phase of pilot program: 2021–2023. In 2025, the LCS will be fully implemented.	Yes (National Pilot LCS Program completed)	Ages 50–74; long-term heavy smokers (≤20 pack-yrs, quit ≤ 15 yrs)	Annual (during 3-year pilot)	Public/EU (EU Social Fund pilot)	[40,41]
Portugal	No	-	-	-	-	-	[42]
Romania	No	-	Implementation of the LCS program in the near future.	-	-	-	[43]
Slovakia	No	-	No (pilot project planned)	Pilot project. Age ≥ 50; High-risk smokers (≤20 pack yrs, quit ≤ 15 yrs)	-	-	[44]
Slovenia	No	-	No	-	-	-	[45]
Spain	Yes (pilot studies for LCS are ongoing (CASSANDRA ^9^) and contribute to the SOLACE ^5^ project)	CASSANDRA: 2023–2028	Yes (multi-region pilot studies, e.g., CASSANDRA ^9^ network)	Ages 50–75 with heavy smoking history; High-risk group (>20 pack yrs, quit ≤ 15 yrs)	-	Public (regional health systems & research networks)	[46,47]
Sweden	No (small single-center pilot studies for LCS are ongoing in Stockholm; pilot studies are planned within the northern, western and southern healthcare regions.)	-	No	-	-	-	[48]

^1^ **NELSON**: Nederlands–Leuvens Longkanker Screening Onderzoek; ^2^ **IMPULSION**: Implémentation du dépistage du cancer Pulmonaire en population; ^3^ **G-BA**: Gemeinsamer Bundesausschuss; ^4^ **HUNCHEST** (^a^ **I**, ^b^ **II**, ^c^ **III**): Hungarian LDCT Lung Cancer Screening program; ^5^ **SOLACE**: Strengthening the screening of Lung Cancer in Europe; ^6^ **RISP**: Rete Italiana Screening Polmonare; ^7^ **NLST**: National Lung Screening Trial; ^8^ **WWRP**: Ogólnopolski Program Wczesnego Wykrywania Raka Płuca; ^9^ **CASSANDRA**: Cancer Screening, Smoking Cessation and Respiratory Assessment.

**Table 2:** Lung cancer screening recommendations in non-EU European countries.

Other Europe Country	LCS Fully Integrated in NS	Years LCS Fully Integrated in NS	LCS Currently In Use	Eligible Group(s) at Risk	Intervals Test of Screening	Funding	References
Norway	No	-	No (only small pilot research studies)	Pilot studies. Age 50–79 yrs; High-risk group	-	-	[49]
Switzerland	No	-	No (pilot projects planned)	The Swiss Lung Cancer Screening Implementation proposes a broad target, e.g., Ages 55–74 yrs; High-risk group (≤30 pack yrs, quit ≤ 15 yrs)	-	-	[50]
United Kingdom	Yes	2023–present	Yes (Targeted Lung Health Check)	55–74 years old, smokers or ex-smokers at high risk (identified from medical records)	Every 2 years (biennially) for those at risk (more frequent follow-ups in the case of suspicious nodules)	Public funding	[51,52]

## Data Availability

The raw data supporting the conclusions of this article will be made available by the authors on request.
